# FGFR1 amplification or overexpression and hormonal resistance in luminal breast cancer: rationale for a triple blockade of ER, CDK4/6, and FGFR1

**DOI:** 10.1186/s13058-021-01398-8

**Published:** 2021-02-12

**Authors:** Silvana Mouron, Luis Manso, Eduardo Caleiras, Jose L. Rodriguez-Peralto, Oscar M. Rueda, Carlos Caldas, Ramon Colomer, Miguel Quintela-Fandino, Maria J. Bueno

**Affiliations:** 1 Breast Cancer Clinical Research Unit, CNIO – Spanish National Cancer Research Center, Melchor Fernandez Almagro, 3, 28029 Madrid, Spain; 2 Medical Oncology Department, Hospital Universitario 12 de Octubre, Madrid, Spain; 3 Histopathology Unit, CNIO, Madrid, Spain; 4 Pathology Department, Hospital Universitario 12 de Octubre, Madrid, Spain; 5 Cancer Research UK Cambridge Institute and Department of Oncology, Li Ka Shing Centre, University of Cambridge, Cambridge, UK; 6 Department of Medicine, Universidad Autonoma de Madrid, Madrid, Spain; 7 Medical Oncology Department, Hospital Universitario La Princesa, Madrid, Spain; 8 Endowed Chair of Personalized Precision Medicine, Universidad Autonoma de Madrid – Fundación Instituto Roche, Madrid, Spain; 9 Unidad de Investigación Clínica y Ensayos Clínicos (UICEC) of Hospital Universitario de La Princesa, Plataforma SCReN (Spanish Clinical Research Network), Instituto de Investigación Sanitaria La Princesa (IP), Madrid, Spain; 10 Medical Oncology Department, Hospital Universitario de Fuenlabrada, Madrid, Spain; 11 Medical Oncology Department, Hospital Universitario Quiron Pozuelo, Madrid, Spain

**Keywords:** FGFR1 overexpression, FGFR1 amplification, Luminal breast cancer, Rogaratinib, Palbociclib

## Abstract

**Background:**

FGFR1 amplification, but not overexpression, has been related to adverse prognosis in hormone-positive breast cancer (HRPBC). Whether FGFR1 overexpression and amplification are correlated, what is their distribution among luminal A or B HRPBC, and if there is a potential different prognostic role for amplification and overexpression are currently unknown features. The role of FGFR1 inhibitors in HRPBC is also unclear.

**Methods:**

FGFR1 amplification (FISH) and overexpression (RNAscope) were investigated in a *N* = 251 HRPBC patients cohort and the METABRIC cohort; effects on survival and FISH-RNAscope concordance were determined. We generated hormonal deprivation resistant (LTED-R) and FGFR1-overexpressing cell line variants of the ER+ MCF7 and T47-D and the ER+, FGFR1-amplified HCC1428 cell lines. The role of ER, CDK4/6, and/or FGFR1 blockade alone or in combinations in Rb phosphorylation, cell cycle, and survival were studied.

**Results:**

FGFR1 overexpression and amplification was non-concordant in > 20% of the patients, but both were associated to a similar relapse risk (~ 2.5-fold; *P* < 0.05). FGFR1 amplification or overexpression occurred regardless of the luminal subtype, but the incidence was higher in luminal B (16.3%) than A (6.6%) tumors; *P* < 0.05. The Kappa index for overexpression and amplification was 0.69 (*P* < 0.001). Twenty-four per cent of the patients showed either amplification and/or overexpression of FGFR1, what was associated to a hazard ratio for relapse of 2.6 (95% CI 1.44–4.62, *P* < 0.001). In vitro, hormonal deprivation led to FGFR1 overexpression. Primary FGFR1 amplification, engineered mRNA overexpression, or LTED-R-acquired FGFR1 overexpression led to resistance against hormonotherapy alone or in combination with the CDK4/6 inhibitor palbociclib. Blocking FGFR1 with the kinase-inhibitor rogaratinib led to suppression of Rb phosphorylation, abrogation of the cell cycle, and resistance-reversion in all FGFR1 models.

**Conclusions:**

FGFR1 amplification and overexpression are associated to similar adverse prognosis in hormone-positive breast cancer. Capturing all the patients with adverse prognosis-linked FGFR1 aberrations requires assessing both features. Hormonal deprivation leads to FGFR1 overexpression, and FGFR1 overexpression and/or amplification are associated with resistance to hormonal monotherapy or in combination with palbociclib. Both resistances are reverted with triple ER, CDK4/6, and FGFR1 blockade.

**Supplementary Information:**

The online version contains supplementary material available at 10.1186/s13058-021-01398-8.

## Introduction

Hormone receptor-positive (HR+) breast cancer accounts for up to 2/3 of the new breast cancer cases. In the long term, more than 20% of HR+ breast cancer patients ultimately die from their disease after developing distant relapse [1, 2]. From the molecular landscape point of view, HR+ breast cancer is a highly heterogeneous disease [3–5], although in the clinical setting, it is commonly classified as luminal A or luminal B (herein Lum-A and Lum-B) based on either the Ki67 replicative fraction [6, 7] or gene-based recurrence scores [7–13].

The relapse pattern of Lum-A and Lum-B cancers (usually several years after the completion of hormonal therapy in the former [1, 2, 14], and more commonly during or shortly after adjuvant hormonal treatment in the latter [14]) suggests that resistance to hormonal therapy plays a key role in the relapse. Hormonal resistance is a complex phenotype that can be caused by different molecular alterations, reviewed elsewhere [15, 16]. One of these factors is the amplification of fibroblast growth factor receptor 1 (FGFR1) [17]. Retrospective studies suggest that 10–15% of primary HR+ breast cancer patients harbor this amplification [3, 17, 18]. FGFR1 amplification has been linked to ligand-dependent and ligand-independent increases in MAPK and Pi3K activation and resistance to hormonal inhibitors [17, 19–21]. Selective FGFR inhibitors (1–4) have shown activity in tumors with FGFRs mutations, amplifications, or fusions [19, 22–25]. The role of FGFR inhibitors in breast cancer, however, is yet unclear [26–29]. Preclinical and clinical data suggest limited single-agent efficacy [24, 29, 30]. There is considerable preclinical evidence regarding the additive effects in combination with hormonal blockade [17, 20], but this evidence has not translated into strong clinical activity [26, 27]. With the current number of available options for advanced HR+ breast cancer [31–38], the development of FGFR inhibitors will be challenging unless a biomarker that narrows down the population that will benefit from FGFR1 blockade and a specific therapeutic niche are determined.

The correlation between amplification and overexpression and the prognostic/predictive role of overexpression of FGFR1 are less clear than those for that human epidermal growth factor 2 (HER2), which is amplified or overexpressed in a similar number of breast cancer cases [39, 40]. Anecdotal studies have reported on FGFR1 overexpression in breast cancer cohorts [41–43], but a standardized test for diagnosing overexpression has been lacking so far. In addition, its prognostic role is currently unknown. Whether amplification and/or overexpression have a distinct distribution among Lum-A and Lum-B patients is also unknown.

In this work, we studied in the incidence and prognostic role of amplification and overexpression of FGFR1 in a cohort of Lum-A and Lum-B HR+ breast cancer patients. FGFR1 mRNA overexpression was determined with a recently developed reproducible assay termed RNAscope [44, 45]. We show that up to 25% of HR+ breast cancer cases harbor amplification and/or overexpression of FGFR1. Both features imply a high risk of relapse. In vitro*,* we found that prolonged hormonal deprivation leads to FGFR1 overexpression and that FGFR1-overexpressing models are insensitive to the combination of fulvestrant plus palbociclib, maintaining a resilient retinoblastoma protein (Rb) phosphorylation and active cell cycle despite the double blockade. FGFR1-amplified or -overexpressing models treated with hormones plus palbociclib were fully eradicated only when rogaratinib (a pan-FGFR1-4 inhibitor that displays activity in different tumors with diverse molecular alterations in FGFR1-4) [46, 47] was added.

## Patients and methods

### Patients

Female patients with a diagnosis of primary, non-metastatic breast cancer with expression of estrogen and/or progesterone receptor > 1% and lack of HER2 amplification diagnosed between January 2001 and December 2002 at Hospital 12 de Octubre were eligible for this study (H12O cohort). The data cutoff for the follow-up of patients was 10 years later (2012), although some patients discontinued clinical follow-up earlier and thus were lost-to-follow-up. The study protocol was approved by the Institutional Review Board of Hospital 12 de Octubre (Ref: 11/137). Access to the METABRIC dataset was granted by Drs. Rueda and Caldas.

### Fluorescence in situ hybridization (FISH) determination and RNAscope

FISH chromosome enumeration probes specifically recognizing FGFR1 were purchased from ZytoVision (ZytoLight SPEC FGFR1/CEN8 Dual Color Probe). FISH analyses were performed according to the manufacturers’ instructions. FISH images were captured using a CCD camera (Photometrics SenSys camera) connected to a PC running the Zytovision image analysis system (Applied Imaging Ltd., UK). Signals were counted in at least 200 cells using the appropriate filters. Results were expressed as the ratio of gene signal to centromere (control) using the following ratios: FISH ratio lower than 1.8 indicates no gene amplification (negative), a ratio higher than 2.2 as gene amplification (positive), and a ratio between 1.8 and 2.2 as equivocal cases. The gene/chromosome copy number alterations were also recorded in the cells as four gene and control signals as moderate polysomy and more than four gene and control signals as high polysomy.

Regarding RNAscope, tissue samples were fixed in 10% neutral buffered formalin (4% formaldehyde in solution), paraffin-embedded and cut at 4 μm, mounted in superfrost®plus slides, and dried overnight. RNAscope staining method was performed in an automated immunostaining platform (Ventana Discovery ULTRA, Roche). Antigen retrieval was first performed with the appropriate buffer and protease (RNAscope VS Universal Sample Prep ReagentV2, 323740, ACD), and endogenous peroxidase was blocked (peroxide hydrogen at 3%). Then, slides were incubated with the human FGFR1 probe, transcript variant 1, mRNA (ACD, 310079). Slides were then incubated with the corresponding Probe Amplification kit (RNAscope VS Universal HRP Detection Reagent, 323210, ACD), conjugated with horseradish peroxidase and reaction was developed using 3, -diaminobenzidinetetrahydrochloride (DAB Detection Kit, 760-224, Ventana, Roche); nuclei were counterstained with Hematoxylin II and slides were mounted. Positive control sections were included for each staining run using Positive Control Probe_Hs-PPIB (313,909, ACD). Samples were acquired and digitalized using the AxioScan.Z1 system (Zeiss). Digitalized images were analyzed with the ZEN 2.3 lite software (Zeiss), and tumoral areas were categorized in the different scores: score 0 (no staining or < 1 dot/10 cells), score 1 (1–3 dots/cell), score 2 (4–9 dots/cell and none or very few are in clusters), score 3 (10–15 dots/cell and < 10% dots are in clusters), and score 4 (> 15 dots/cell and > 10% dots are in clusters). Scores of 3 and 4 were considered RNAscope-positive.

### In vitro experiments

MCF7, T47-D, and HCC1428 cells were acquired from the American Type Culture Collection (ATCC). Cells were maintained following the ATCC recommendations and routinely tested for mycoplasma using the MycoalertTM Mycoplasma Detection Kit (Lonza). Cell lines were authenticated every 6 months using short-tandem repeat profiling. Cell line clones resistant to estrogen deprivation were generated following the method described by Martin et al [48, 49]. Briefly, the method consisted of weekly passage and culture of cells in medium containing 10% dextran charcoal-stripped (DCC) fetal bovine serum (FBS) (Sigma) instead of full FBS, which removes steroids. The medium was changed every 2–3 days for 2 years until acquisition of the LTED-R phenotype.

A retroviral vector for human FGFR1 overexpression (pWZL_Neo_Myr_Flag_FGFR1) was purchased from Addgene (Cat#20486). To generate stably transduced human cells (MCF7 and T47-D), 4 μg of FGFR1-encoding plasmid was co-transfected with 3 μg of pCL-Ampho retrovirus packaging vector (Imgenex, Cat#10046P) into HEK 293 T cell using Lipofectamine 2000 (Thermo Fisher). HEK 293T medium was changed 24 h post-transfection. Virus-containing supernatants were harvested 48 h post-transfection, passed through a 0.45-mμ filter, diluted 1:4, and applied to target cells with 8 μg/mL polybrene (Sigma Aldrich). Transduced cells were selected in 750 μg/mL neomycin.

Colony-formation assays were conducted as follows: breast cancer cell lines were seeded at densities of 1500 (MCF7, MCF7-FGFR1, MCF7-LTED-R, T47-D, T47-D-FGFR1, and T47-D-LTED-R) and 4000 (HCC1428 and HCC1428-LTED-R) cells per well in 12-well plates. After overnight incubation, medium was replaced with fresh medium with either vehicle (control) or drugs. Rogaratinib and palbociclib were obtained from Bayer Inc. and Pfizer Inc. respectively, through material transfer agreements. Fulvestrant was purchased from MedChem Express. Media and drugs were refreshed every 3–4 days. After 10 days of culture, cells were fixed and stained with 0.1% (w/v) crystal violet in 10% (v/v) ethanol. All experiments were performed at least in triplicate. The well area covered by colonies (colony area intensity) was quantified automatically from flatbed scanner-acquired images of colony assays conducted in multi-well plates using the ImageJ software [50].

To determine the inhibitory concentration of 50% (IC50) of fulvestrant and palbociclib in MCF7, T-47D, and HCC1428 cell lines, clonogenic survival assays were performed. Cells were exposed to a concentration range of fulvestrant and palbociclib. The IC50 values were derived by a sigmoidal dose-response (variable slope) curve using GraphPad Prism software version 5.04. Values shown in Supplemental Table 2 represent the mean of three independent experiments.

Regarding cell-cycle assays, cells were pre-treated with drugs or vehicle for 48 h and then 10 μM BrdU was added to the medium for 30 min before harvesting. Fixed cells were treated with 2 M HCl for 20 min, and BrdU was immunolabeled with FITC-conjugated anti-BrdU (Cat. 556028, BD Pharmigen™). For DNA-content analysis, cells were fixed in 70% ethanol, washed in PBS, and stained with 50 μg/ml propidium iodide (Sigma) in the presence of 10 μg/ml RNase A (Qiagen). Flow cytometry data were acquired in a FACSCanto cytometer (BD Biosciences) and analyzed with FlowJo software (Tree Star Inc.).

The following primary antibodies were used for immunoblots: phospho-Rb (Ser780), Rb (clone D20), FGFR-1 (clone D8E4), pFRS2 (Tyr196), pFRS2 (Tyr436) (all from Cell Signaling Technology), CCND1 (clone A12, Santa Cruz), FRS2 (Proteintech), and pFGFR1 (Tyr653/654) (Invitrogen). Vinculin and β-Actin (clone AC-15) (Sigma) were used as a loading control. Membranes were incubated with appropriate peroxidase-conjugate secondary antibodies (Sigma, St Louis, MO, USA). Bands were visualized by the enhanced chemiluminescence (ECL) method (Lumi-LightPlus detection kit; Roche). FGFR1, 2, 3, and 4 expression was performed using TaqMan technology using TaqMan® Fast Advanced Master Mix (Applied Biosystems) in a QuantStudio™ 6 Flex Real-Time PCR system (Applied Biosystems). The probes from Applied Biosystems were used for FAM/MGB-NFQ FGFR1 (Hs00241111_m1), FAM/MGB-NFQ FGFR2 (Hs01552918_m1), FAM/MGB-NFQ FGFR3 (Hs00179829_m1), FAM/MGB-NFQ FGFR4 (Hs01106910_g1), and FAM/MGB-NFQ TFRC (Hs00951083_m1), as housekeeping genes.

### Statistics

Survival functions were computed using the Kaplan-Meier estimator. Differences in survival between groups were assessed using the Log-Rank test. The hazard ratio for relapse risk for the different tested subgroups determined by the luminal A or B status and/or FGFR1-overexpression/amplification were obtained fitting a Cox’s proportional hazards model. The concordance analysis between FISH and RNAscope was performed with the Kappa index. The correlation analysis to evaluate the association between FGFR1 overexpression and FGFR1 gene copy number was performed with the Pearson test. The number of amplified cases among luminal A and B cases were compared with Fisher’s exact test. All tests were two-tailed and were performed with the SPSS Statistics V.19.0 software.

## Results

### FGFR1-amplified and/or FGFR1 mRNA-overexpressing hormone-positive cases have an adverse clinical course

**Fig. 1:**
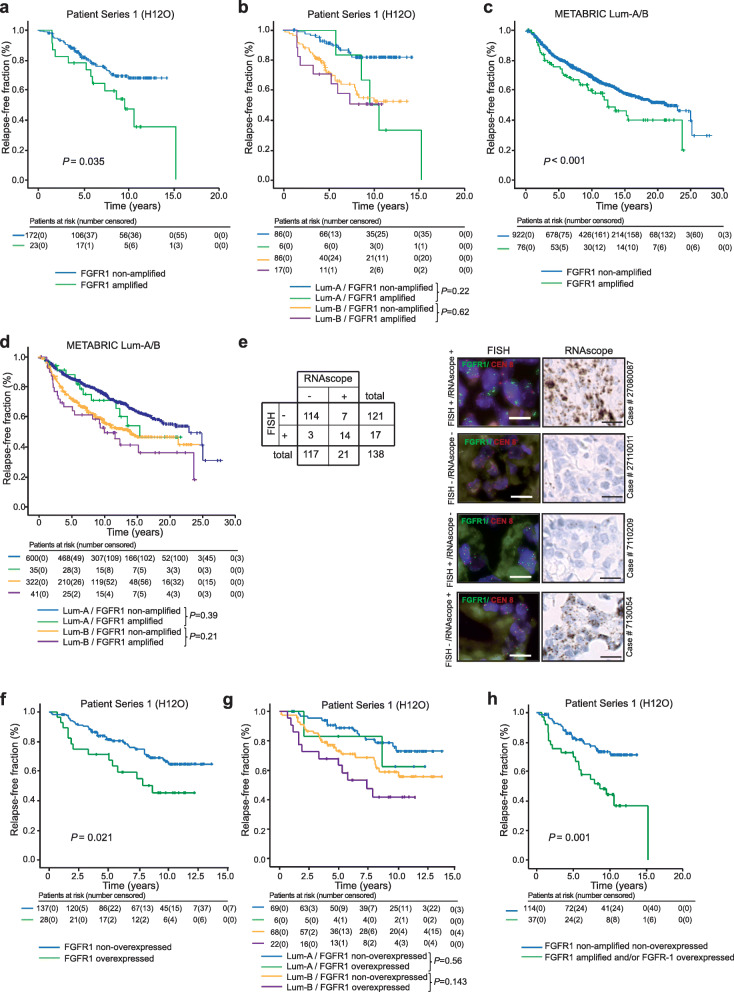
Concordance between FGFR1 amplification and overexpression and prognostic impact among hormone-positive breast cancer patients. Kaplan-Meier curves representing the impact of FGFR1-amplficiation disease relapse risk in the general HR+ breast cancer cohort (**a**) or split according to the Lum-A or Lum-B subtypes (**b**) in the discovery set (H12O series) or in the METABRIC Lum-A/B cohort (**c**) and (**d**). **e** Left panel: concordance between the result of FGFR1 FISH and RNAscope tests. Right panel: examples of concordant results (positive or negative in both tests) or non-concordant results (positive/negative or vice versa) in different cases of the H12O series. **f**, **g** Same as **a** and **b** according to RNAscope positivity. **h** Kaplan-Meier curve for distant relapse of patients with FGFR1 amplification and/or FGFR1 mRNA overexpression compared to non-amplified, non-overexpressed cases. Patients at risk are stated under each Kaplan-Meier curve. Vertical ticks represent censoring events

We first studied the role of FGFR1 amplification (ratio FGFR1/centromere > 2.2) in a retrospective series of *N* = 251 patients (H12O cohort). The clinical and demographic characteristics of this cohort are listed in Table 1. Patients with FGFR1 amplification (11.8%; *N* = 23/195; fluorescence in situ (FISH) did not yield a valid result in 56 patients) displayed a significantly worse prognosis. Median relapse-free survival time was not reached in FGFR1-non-amplified patients (average 11.2 years) compared to a median relapse-free survival time of 9.5 years (average 9.3 years) in patients with FGFR1 amplification (Log-Rank *P* = 0.035; Fig. 1a). Since it has been proposed that FGFR1 might confer an adverse prognosis only in cases of “high amplification” [29], we also tested the prognostic effect in patients harboring a ratio FGFR/centromere of > 4 and > 6 (Supplemental Figure 1); the number of amplified patients following such definitions were lower and thus the significance was preserved only for the first case.

**Table 1:** Clinical and demographic characteristics

Characteristic	H12O series (***N*** = 251 patients)	METABRIC (hormone-positive, Lum-A or Lum-B subset) (***N*** = 998 patients)
**Age (median, range)**	54.0 (20.2–91.0)	63.9 (26.3–90.4)
**Tumor size**		
T1	126 (50.2%)	440 (44.0%)
T2	96 (38.2%)	519 (52.0%)
T3	23 (9.1%)	36 (3.6%)
T4	6 (2.4%)	N/A**
N/A	0 (0%)	5 (0.5%)
**Nodal status**		
N0	108 (43.1%)	540 (54.0%)
N1	85 (33.9%)	313 (31.3%)
N2	38 (15.1%)	105 (10.5%)
N3	20 (7.9%)	40 (4.0%)
**Grade**		
G1	63 (25.1%)	127 (12.7%)
G2	126 (50.2%)	502 (50.3%)
G3	62 (24.7%)	326 (32.6%)
N/A	0 (0%)	44 (4.4%)
**Lum-A/B (defined by Ki67% staining)**		
< 15% (Lum-A)	124 (49.4%)	N/A
> 14% (Lum-B)	127 (50.6%)	
**Lum-A/B (defined by PAM-50)**		
Lum-A	N/A	635 (63.6%)
Lum-B		363 (36.4%)
**Adjuvant/neoadjuvant chemotherapy**		
**No**	63 (25.1%)	920 (92.2%)
**Yes**	188 (74.9%)	78 (7.8%)
CMF or capecitabine	38 (15.1%)	14 (1.4%)
Anthracycline based	87 (34.7%)	38 (3.8%)
Taxane based	63 (25.1%)	4 (0.4%)
Other	0 (0%)	22 (2.2%)
**Adjuvant hormonal therapy**		
No	13 (5.2%)	273 (27.4%)
Yes	238 (94.8%)	725 (72.6%)
FGFR1 amplified*	195/251 available (77.7%)	998/998 available (100%)
No	172 (88.2%)	922 (92.4%)
Yes	23 (11.8%)	76 (7.6%)
**FGFR1 RNAscope positivity**	165/251 available (65.7%)	
Negative (0, 1+, or 2+)	137 (83%)	N/A
Positive (3+ or 4+)	28 (17%)	N/A
**Relapse**		
No	169 (73.3%)	627 (62.8%)
Yes	82 (32.7%)	370 (37.1%)
N/A	0 (0%)	1 (0.1%)

*FGFR amplification was determined in the H12O series by FISH in a tissue microarray. Conversely, in the METABRIC series, it was determined by CGH arrays. FISH data were not available in 56 cases; thus, positive/negative cases are shown in relative percentage to the available cases

**In the METABRIC database, primary tumor is coded by size in millimeters. What qualifies a primary tumor as T4 is the invasion of the chest wall and/or skin and/or presence of inflammatory carcinoma, regardless of the tumor size in millimeters. Thus, the number of T4 tumors in this series is actually unknown, although the percentage of T4 tumors in routine clinics is generally low

According to Ki67 staining, the H12O series was composed by *N* = 124 (49.4%) Lum-A and *N* = 127 (50.6%) Lum-B cases; FGFR-1 was amplified in a significantly higher percentage of Lum-B (16.3%; *N* = 17/103; FISH not valid in 24 patients) than Lum-A (6.6%; *N* = 6/92; FISH not valid in 32 patients) patients (*P* = 0.044). The FGFR1 to CEN8 FISH ratio, Ki67 value, luminal status, and survival information are listed in Supplemental Table 1. Whereas the hazard ratio for relapse conferred by FGFR1 amplification was statistically significant (HR = 1.96; 95% CI 1.031–3.72; *P* = 0.038), the significance was not preserved when the cohort was split by the luminal subtype. Median relapse-free survival times were in Lum-A/FGFR1-non-amplified, Lum-A/FGFR1-amplified, Lum-B/FGFR1-non-amplified, and Lum-B/FGFR1-amplified, respectively: not reached (average 12.5 years), 9.5 years (average 10.8 years), not reached (average 9.4 years), and not reached (average 7.3 years). The overall Log-Rank *P* value for the relapse-free time comparison among the 4 groups was < 0.001; the pairwise comparisons were as follows: Lum-A/FGFR1-non-amplified versus Lum-A/FGFR1-amplified: *P* = 0.22; hazard ratio for relapse 3.4 (95% CI 1.12–10.57; *P* = 0.031); Lum-B/FGFR1-non-amplified versus Lum-B/FGFR1-amplified: *P* = 0.62; hazard ratio for relapse: 1.21 (95% CI 0.55–2.64; *P* = 0.62); the Kaplan-Meier curves are shown in Fig. 1b).

We then validated externally our findings in the METABRIC study cohort, constituted by 1992 breast cancer patients, where the intrinsic subtype was determined by gene expression. The cohort is described elsewhere [3] and was constituted by mostly low-risk patients. Of them, 998 cases where either estrogen receptor-positive and/or progesterone receptor-positive, HER2-negative, and either Lum-A or Lum-B (herein, METABRIC Lum-A/B). The main clinical and demographic characteristics of these 998 patients are shown in Table 1. In general, compared to the H12O cohort, these cases displayed more benign characteristics such as older age or more frequent T1/T2 and N0/N1 stages and Lum-A profile. Seventy-six (7.6%) of METABRIC Lum-A/B patients harbored FGFR1 amplification. These patients had an adverse disease course compared to the remainder, with a median relapse-free survival of 12.2 years compared to 21.1 years for the patients without FGFR1 amplification (Log-Rank *P* = 0.029; Fig. 1c).

Six hundred and thirty-five METABRIC Lum-A/B were Lum-A (63.6%) whereas 363 (36.4%) were Lum-B. Akin to the high-risk H12O series, the percentage of FGFR1 amplifications was higher in Lum-B cases (11.1% versus 5.5% in Lum-A cases; *P* = 0.001). The median relapse-free survival for Lum-A/FGFR1-non-amplified, Lum-A/FGFR1-amplified, Lum-B/FGFR1-non-amplified, and Lum-B/FGFR1-amplified subgroups was 22.7, 15.2, 13.6, and 9.8 years, respectively (overall Log-Rank *P* < 0.001). The pairwise comparisons, however, akin the H12O series, lost significance when the series was split by the luminal subtype: regarding Lum-A, the Log-Rank *P* value was 0.39, whereas for Lum-B cases, the Log-Rank P was 0.21; the hazard ratio for relapse were, respectively, 1.27 (95% CI 0.72–2.23) and 1.3 (95% CI 0.85–2.04); Fig. 1d).

We then analyzed FGFR1 RNA expression levels by RNAscope in the H12O cohort. Out of 165 cases with a valid result, 28 of them (17%) were RNAscope positive (score of 3 or 4, herein FGFR1-overexpressed) (Supplemental Table 1). The rate of positivity was higher than the percentage of FGFR1-amplified cases in the H12O (*P* = 0.07) and METABRIC Lum-A/B series (*P* < 0.001). There were more RNAscope-positive cases among Lum-B than Lum-A patients (24.4% versus 8.0%; *P* < 0.001). (Supplemental Table 1). The concordance between positivity in FGFR1 by FISH and RNAscope is depicted in Fig. 1e (it was considered only samples with valuable information for both techniques). The Kappa index for this concordance was 0.69 (*P* < 0.001); examples matching and non-matching results in FISH and RNAscope are shown in Fig. 1e. FGFR1 copy number and FGFR1 RNAscope values also showed a statistically significant positive correlation (Pearson coefficient: 0.61; *P* < 0.001). The effect of FGFR1 overexpression in distant relapse was similar than that of FGFR1 amplification: the median relapse-free survival time was not reached for patients without overexpression (average 10.69 years) compared to 8.6 years (average 7.7) for the patients with FGFR1 overexpression (Log-Rank *P* = 0.021; Fig. 1f). The hazard ratio for relapse conferred by FGFR1 overexpression was 2.02 (95% CI 1.096–3.73, *P* = 0.024). The median relapse-free survival times were in Lum-A/FGFR1 non-overexpressed, Lum-A/FGFR1-overexpressed, Lum-B/FGFR1 non-overexpressed, and Lum-B/ FGFR1-overexpressed, respectively: not reached (average: 11.6 years), not reached (average: 9.7 years), not reached (average: 9.7 years), and 7.3 (average: 6.9 years). The pairwise comparisons (Lum-A/FGFR1 non-overexpressed versus Lum-A/FGFR1-overexpressed; and Lum-B/FGFR1 non-overexpressed versus Lum-B/ FGFR1-overexpressed), however, were not significant: *P* = 0.56 and *P* = 0.143, respectively.

Finally, if we consider together either amplification and/or overexpression of FGFR1, patients that harbored any of the two alterations (24.5% of the patients, *N* = 37/151) displayed a significantly worse prognosis compared to patients that did not have any (Fig. 1h; median relapse free survival of 8.6 years (average 8.8) versus not reached (average 11.2); Log-Rank *P* < 0.001; the hazard ratio for relapse was 2.6 (95% CI 1.44–4.62; *P* < 0.001)).

### Prolonged hormonal deprivation leads to increased FGFR1 transcriptional levels and cross-resistance with fulvestrant

**Fig. 2:**
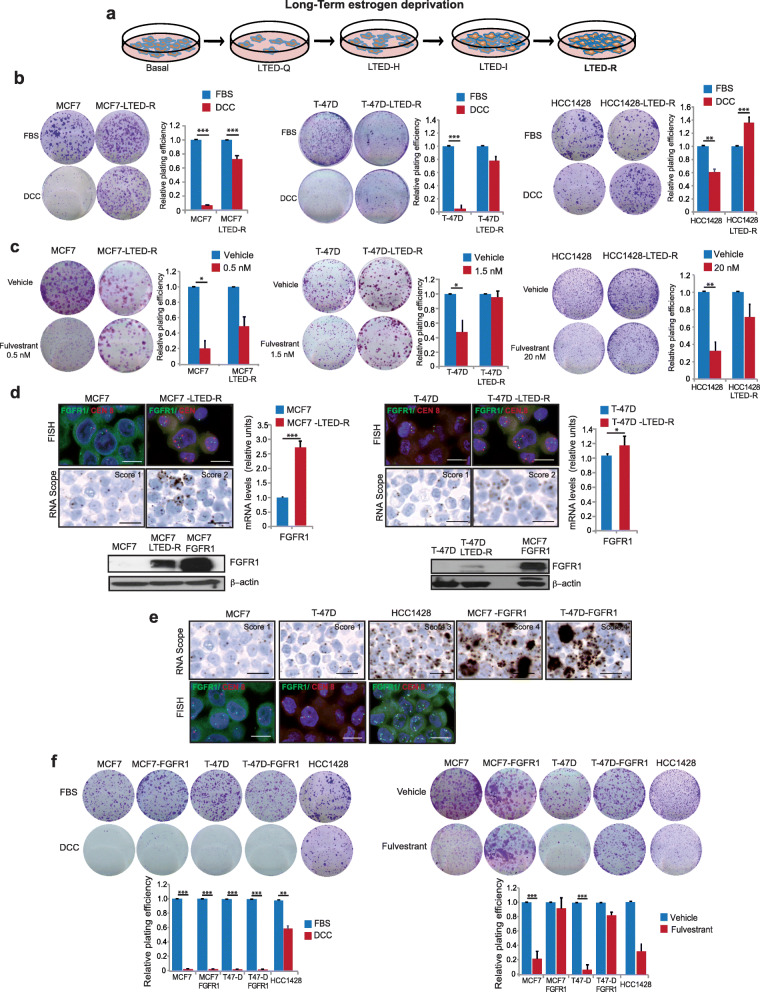
Prolonged hormonal deprivation selects for cells with high FGFR1 mRNA levels which are resistant hormonal deprivation and fulvestrant. **a** Cell lines were exposed to estrogen deprivation by culturing and passaging them for > 24 months in regular culture media supplemented with 10% dextran charcoal-stripped (DCC) fetal bovine serum (FBS), what deprives FBS from estrogens. Cells undergo 4 progressive stages from the basal status (hormone-dependent) until the acquisition of resistance during a ~ 2-year process: quiescent long-term estrogen deprivation (LTED-Q; most cells have died and a number of cells remain but do not replicate), hypersensitive to estrogens (LTED-H; cells still do not replicate but if they are exposed to estrogens they replicate fast), estrogen independent (LTED-I; they are able to grow and replicate in absence of hormones but much slower than the parental), and hormonal-resistant (LTED-R; they behave in absence of hormones akin parental cells in presence of hormones). **b** Representative pictures of colony assays and quantification (relative plating efficiency—RPE) of MCF7, T-47D, and HCC1428 and their LTED-R variants in full or DCC media. For each cell line, RPE is depicted relatively to its own RPE in full media. **c** Same as in **b**, comparing vehicle versus fulvestrant at the depicted concentrations. **d** FGFR1 genetic (assessed by FISH—upper pictures), transcriptomic (assessed by RNAscope—lower pictures—and RTPCR—charts) and protein status (determined by western blot) of parental and LTED variants of MCF7 and T-47D. MCF7-FGFR1 is included in the western blot for control purposes. In FISH pictures: green dots: FGFR1 probe; red dots: chromosome 8 centromere probe. **e** FGFR1 copy number (FISH, above) and mRNA levels (RNAscope, below) in parental MCF7 and T-47D cell lines or mRNA levels (RNAscope) for engineered to overexpress FGFR1. The amplified HCC1428 cell lines are included for control purposes. **f** Colony assays and RPE of the same cell lines shown in **e** exposed to estrogen deprivation or fulvestrant (MCF7 cell lines, 0.5 nM; T47-D cell lines, 1.5 nM and HCC1428 cell line, 20 nM), indicating how FGFR1 mRNA high cell lines are resistant to fulvestrant regardless of amplification. Error bars: standard error. ***P < 0.001; **P < 0.01; *P < 0.05. Scale bars: FISH, 10 μm; RNAscope, 20 μm

The mainstay of treatment for Lum-A/B tumors in the adjuvant setting is hormonal blockade. The majority of Lum-A/B patients receive 5–10 years of hormonal blockade in the adjuvant setting. Aromatase inhibitors are the most frequently prescribed agents, and they reduce the available estrogens in the organism to non-detectable levels. FGFR1 signaling has been implicated in resistance to hormonal inhibition [3, 17–21]; in addition, hormonal deprivation has been related to FGFR1 overexpression [20]. In order to mimic this scenario in vitro, and to ascertain its functional role, we exposed 2 FGFR1-non-amplified (MCF7, T-47D) and 1 FGFR1-amplified (HCC1428) breast cancer cell line to prolonged hormonal deprivation (> 2 years) (Fig. 2a). The three cell lines acquired the long-term estrogen-deprivation resistant phenotype (LTED-R). MCF7 and T-47D parental cell lines had a > 90% decrease in relative plating efficiency (RPE) whereas HCC1428 (FGFR1-amplified) had around a 40% decrease in RPE in 10-days colony assay experiments in estrogen-free DCC media. Conversely, MCF7-LTED-R, T-47D-LTED-R, and HCC1428-LTED-R variants showed very similar plating efficiency rates in estrogen-free and -full media (Fig. 2b). Interestingly, the LTED-R phenotype was cross-resistant to fulvestrant (a selective estrogen receptor degrader which is a commonly used agent in combination with CDK4/6 inhibitors upon metastatic relapse [31–34]), as shown by the limited effects in plating efficiency observed for the three LTED-R variants compared to the parental cell lines (Fig. 2c). As it can be observed, for FGFR1-amplified cell line HCC1428, a 10-fold higher fulvestrant concentration than that required for MCF7 and T-47D cell lines was required to have a suppressive effect on parental cell line (Fig. 2c).

Prolonged hormonal deprivation has been related with FGFR1 overexpression [20], but whether this reflects the selection of previously existing clones or is the result of a new genomic event is currently unknown. Interestingly, both MCF7-LTED and T-47D-LTED variants showed a polysomic (but not amplified) FGFR1 locus in FISH, increased FGFR1 mRNA levels by RT-PCR, positive RNAscope staining, and increased FGFR1 protein levels compared to the parental cell lines (Fig. 2d). These data suggest that cell line subclones with increased FGFR1 genetic and transcriptomic levels were positively selected during the LTED phenotype acquisition experiment. Whether these clones were previously present (“persistence”) and expanded through the 2-year selection process, or appeared de novo by mutational processes, it is difficult to ascertain with the current data. However, the limited cellular replication during the acquisition of the LTED-R phenotype (Supplemental Figure 2A) and the presence of a minor number of polysomic cells in the parental cell lines (Supplemental Figure 2B) may suggest that the persistence and expansion of resistant clones explains the phenotype.

Finally, we engineered the non-FGFR1-amplified cell lines T-47D and MCF7 to overexpress FGFR1 (without amplification). The comparative FISH and RNAscope of FGFR1 among the two FGFR1-low cell lines (T-47D, MCF7), their two FGFR1-overexpressing variants (T-47D-FGFR1 and MCF7-FGFR1; unchanged FISH but increased RNAscope versus the parental), and the FGFR1-amplified HCC1428 cell line were showed in Fig. 2e and Supplemental figure 2C. Although engineered FGFR1-overexpression did not seem to confer primary resistance to estrogen deprivation in MCF7 and T-47D cell lines (Fig. 2f, left panel), it was clearly related to primary resistance to fulvestrant (Fig. 2f, right panel; in both panels the primary FGFR1-amplified HCC1428 is shown for control purposes). The IC50 for fulvestrant on each cell line is shown in Supplemental Table 2. Taken together, these data suggest that the increase of FGFR1 mRNA levels, regardless of the amplification status, is associated with hormonal resistance. Prolonged estrogen deprivation leading to increased FGFR1, or primarily amplified FGFR1, seems to be related to DCC and fulvestrant resistance. The fact that engineered FGFR1 overexpression led to resistance to fulvestrant but not to DCC suggests that during the acquisition of the LTED-R phenotype, other events may mediate in the process. Since fulvestrant is commonly prescribed in combination with a cell cycle inhibitor upon progression to aromatase inhibitors [33, 34] (i.e., the scenario mirrored by LTED-R variants), we next explored the sensitivity of these variants to CDK4/6 inhibition and evaluated the blockade of FGFR1 as a potential way to sensitize them to fulvestrant plus palbociclib.

### Hormone-resistant, FGFR1-amplified, or overexpressed cell lines are resistant to the double but sensitive to the triple blockade

**Fig. 3:**
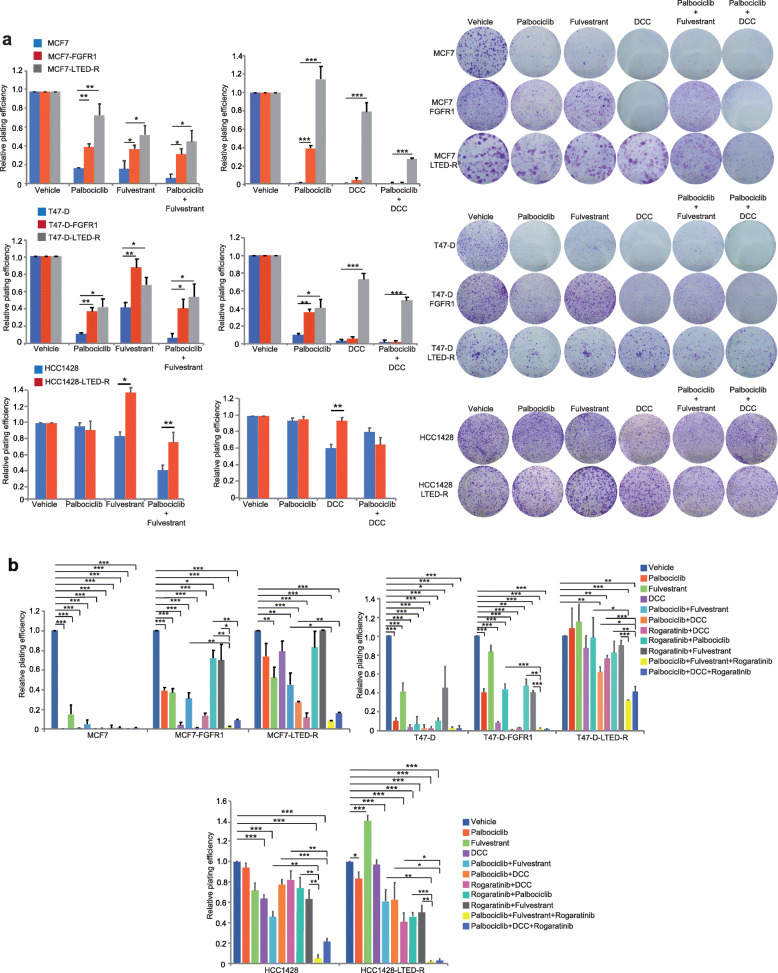
Triple ER, CDK4/6, and FGFR1 blockade reverts resistance in FGFR1-positive models. **a** Relative plating efficiency (left) and example of colony assays (right) of parental MCF7, T47-D, and FGFR1-amplified HCC1428 or their FGFR1-overexpressing or LTED-R variants in response to vehicle, palbociclib (MCF7 cell lines, 50 nM; T47-D and HCC1428 cell lines, 100 nM), fulvestrant (MCF7 cell lines, 0.5 nM; T47-D cell lines, 1.5 nM and HCC1428 cell lines, 5 nM), DCC, palbociclib plus fulvestrant, or palbociclib plus DCC. **b** In the same model as in **a**, the observed resistance against palbociclib + fulvestrant/DCC was reverted by adding the FGFR1 inhibitor rogaratinib (1 μM), as evidenced by the relative-plating efficiency data. Statistically significant difference between either vehicle and all combinations or double and triple combinations are shown. Error bars: standard error. ***P < 0.001; **P < 0.01; *P < 0.05

Currently, the standard-of-care for metastatic HR+ breast cancer is the combination of either an aromatase inhibitor or fulvestrant with a CDK4/6 inhibitor, both in the first and second line [31–36]. Thus, we tested the sensitivity to hormonal deprivation (DCC) or fulvestrant plus palbociclib of the primary FGFR1-amplified cell line, the engineered FGFR1-overexpressing cell lines, and the LTEDR variants with high FGFR1 mRNA levels. Compared to the parental cells, FGFR1-overexpressing engineered and to a greater extent LTED-R variants were more resistant to DCC, fulvestrant, palbociclib, and DCC/fulvestrant+palbociclib doublets (Fig. 3a). The primarily amplified cell line HCC1428 and its LTED-R variant were, as expected, resistant to the combinations as well (Fig. 3a). Thus, cell lines with increased FGFR1 copy number or mRNA, primarily or as a result of prolonged hormonal deprivation, are resistant to the combination of hormonal deprivation plus palbociclib. The IC50 for palbociclib in the 7 cell lines is shown in Supplemental Table 2.

We next tested the effects of FGFR1 inhibition with the selective pan-FGFR inhibitor rogaratinib [51]. Rogaratinib effectively inhibited ERK, FGFR1, and FRS2 phosphorylation in response to the FGFR1 ligand bFGF in FGFR1-high or LTED-R cell lines (Supplemental Figure 3). Despite the evidenced pharmacodynamic effect, however, rogaratinib did not show efficacy in monotherapy in any cell line (Supplemental Figure 4). The expression levels of the other rogaratinib targets, FGFR2, 3, and 4, are shown in Supplemental Figure 5. These data are in concordance with published studies that suggest that FGFR inhibitors in breast cancer would have effect only in combination with hormonal blockade [17, 20]. However, the triple combinations of fulvestrant or DCC plus palbociclib and rogaratinib were the only ones that achieved 80–100% suppression of RPE in the colony assays of all models, including the resistant FGFR1-amplified and/or FGFR1 mRNA-high LTED-R variants, FGFR1-overexpressing clones, or native HCC1428 cell lines (Fig. 3b).

**Fig. 4:**
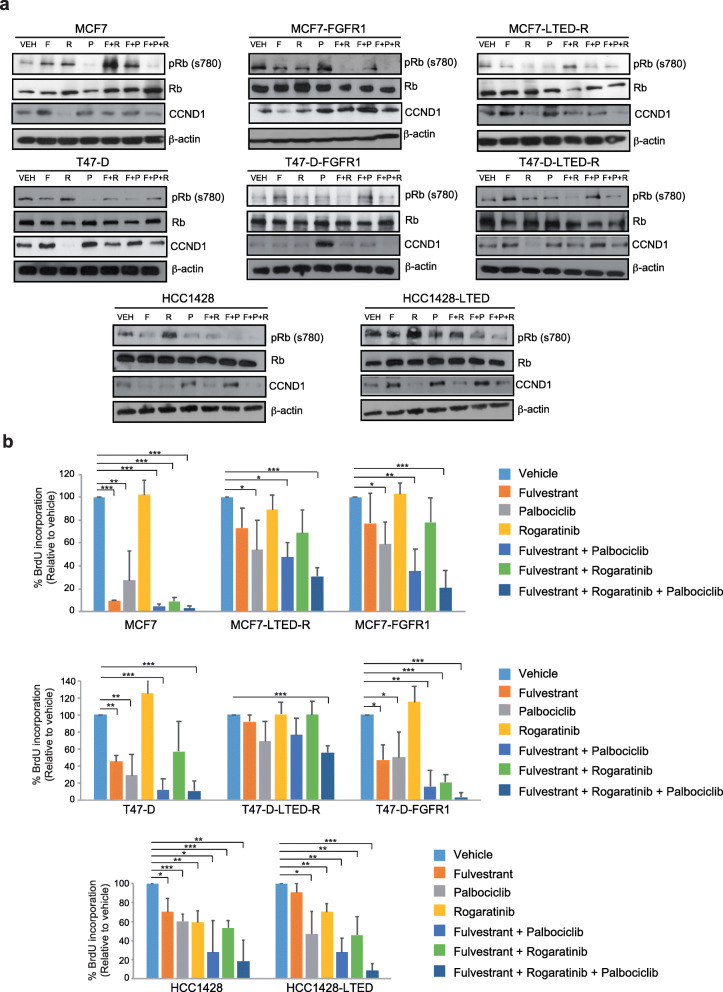
Rb phosphorylation and cell cycle suppression with the triple FGFR1, ER, and CDK4/6 blockade. **a** Western blot showing the phosphorylation (Ser780) of the Rb protein 48 h after treatments. For each cell line (MCF7 or T47D plus their LTED-R and FGFR1-overexpressing variants, and the FGFR1-amplified HCC1428 and its LTED-R variant), 7 conditions were tested: vehicle (VEH), fulvestrant (F), rogaratinib (R), palbociclib (P), fulvestrant plus rogaratinib (F + R), fulvestrant plus palbociclib (F + P), and fulvestrant plus palbociclib plus rogaratinib (F + P + R). Drug concentrations: MCF7 cell lines: F, 0.5 nM; P, 50 nM; R, 1 μM; T-47D cell lines: F, 1.5 nM; P, 100 nM; R, 1 μM; HCC1428 cell lines: F, 5 nM; P, 100 nM; R, 1 μM. Total Rb and beta-actin are shown for control purposes. It can be appreciated that in the FGFR1-high models (either because of engineered overexpression, LTED-R phenotypes, or primary amplification), the maximum suppression is achieved in the rogaratinib-containing combinations. Whereas the doublet (F + R) is highly active in most models, the triplet (F + P + R) is the only one that achieves suppression in all the models. Conversely, wild-type MCF7 and T-47D do not require rogaratinib for suppression, and actually, the greater effect is achieved by palbociclib. CCND1 protein levels showing that the effects on pRb are mediated by activation/inhibition of Cyclin D/CDK4/6 axis. **b** Charts showing BrdU incorporation (S-phase) of the same models and concentrations as in **a**, evidencing the translation of the effects over Rb phosphorylation in the cell cycle. The maximum effects in the FGFR1-high models are achieved by the triplet in all cases. Error bars: standard error. ***P < 0.001; **P < 0.01; *P < 0.05

Epithelial hormone-positive cell lines transduce replicative signals that converge in the phosphorylation and inactivation of Rb by the CDK4/cyclin-D complex, which is then targeted for proteasomal degradation [52, 53]. CDK4/6 inhibitors thus block Rb phosphorylation, which gets stabilized and inhibits the transcription factor E2F, stopping the entry into S-phase [53, 54]. Alternative pathways implicated in sustained Rb phosphorylation in absence of CDK4/6 activity are not well understood; however, the LTED-R and FGFR1-overexpressed cell lines displayed a resilient Rb phosphorylation regardless of hormonal blockade with or without palbociclib (Fig. 4a). It has to be noted that even in MCF7 and T-47D parental cells, the treatment with fulvestrant as a single agent does not suppress pRb levels. In addition, pRb levels are retained upon palbociclib treatment in LTED-R and FGFR1-overexpressing cells, despite the greater effect achieves by palbociclib in parental cells. Although fulvestrant or palbociclib led to some decrease of phosphorylation of Rb, the maximum effect was achieved with either fulvestrant + rogaratinib or fulvestrant + palbociclib + rogaratinib, which led to complete abrogation in several models. Similar effects were observed in the HCC1428 model (Fig. 4a). Previous studies have suggested at least a partial causal role of the FGFR-cyclin D1 axis in resistance to antiestrogens alone and in combination with CK4/6 inhibitors [55]. The reduction in CCND1 protein levels after triple combination treatment revealed that the effects on Rb phosphorylation is mediated by inhibition of Cyclin D/CDK4/6 axis (Fig. 4a).

BrdU incorporation assay performed in the different models, with or without drugs (monotherapy or combinations), revealed that the suppression of Rb phosphorylation actually correlated with the effects observed in cell cycle across the different models, where cells displayed their lowest S-phase fractions in presence of the triplets (Fig. 4b and Supplemental Figure 6). Taken together, our data suggest that HR+ breast cancer cell lines with elevated FGFR1 levels (either because of amplification or increased mRNA levels) require the combination of ER, CDK4/6 and FGFR1 blockade for maximum cell cycle arrest.

## Discussion

In the rapidly evolving field of HR+ breast cancer therapeutics, a key objective is how to maintain sensitivity to hormonal-based therapies for as long as possible, delaying the need for standard cytotoxic chemotherapy. The recent incorporation of cell cycle inhibitors in the first- and second-line metastatic setting has revolutionized the field, doubling the average progression-free survival times of hormonal treatments alone [31–36]. Other strategies targeting the Pi3K [37] or the MTOR [38] pathways have proved successful as well in extending the time during which patients benefit from hormonal blockade. However, multiple signaling pathways are involved in the acquisition of hormonal resistance [15]. The contribution to hormonal resistance varies from one pathway to another, and probably more than one usually co-exist; in addition, the acquisition of the hormonal resistant phenotype is rather a continuum [49] than a “black or white” situation, and is influenced by previous length and type of hormonal exposure. The complex and inter-patient variable genomic landscapes, together with the effects caused by previous drug exposures, will certainly make the field increasingly challenging in order to individually select the best agent combo to extend hormonosensitivity.

On top of the mentioned CDK4/6, Pi3K, and MTOR targets, FGFR1 may be another key signaling axis driving hormonal resistance. Single-agent FGFR1-inhibitors do not show sufficiently high activity in advanced HR+ breast cancer to warrant further development [24, 29, 30]. Combinations of a hormonal agent and an FGFR1-inhibitor show variable activity [26, 27]. Our results advance in the task of finding a therapeutic scenario and a patient sub-population where FGFR inhibitors would deserve clinical investigation: HR+ breast cancer patients with amplification and/or overexpression of FGFR1 upon progression to aromatase inhibitors.

The incidence of FGFR1 amplification has been already described and several studies consistently report a ~ 10% rate among patients with a primary HRPBC, which, in turn, is associated with a 2- to 4-fold higher risk of metastatic relapse [3, 17, 18]. However, the incidence and prognostic role of FGFR1 mRNA overexpression in breast cancer was previously unknown. RNAscope is a technique that can determine overexpression of mRNA in paraffinized samples and has a standardized quantitation method [44, 45]; FGFR1 has not been previously studied in breast cancer by this method. Interestingly, in the H12O series, we found that the hazard ratio for metastatic relapse conferred by FGFR1 mRNA overexpression was virtually the same to that conferred by amplification; however, it affected to a significantly greater number of patients (Fig. 1e, f). In addition, it is worth noting that although most amplified cases tested positive in RNAscope and vice-versa, there was a significant number of patients that did not (Fig. 1e), suggesting that not all tumors with FGFR1 amplification overexpress FGFR1 and that overexpression can be achieved without amplification. In order to detect all patients in which FGFR1 confers an adverse prognosis, FISH and RNAscope should be performed together: the Kaplan-Meier curve shown in Fig. 1h shows that patients that are positive by either technique (or both) have a hazard ratio of relapse > 2.5. This data can only be underscored, since as much as 24.5% of the H12O series tested positive in at least one of the two tests, which represents a greater percentage of breast cancer patients than triple-negative or HER2-positive tumors that could potentially benefit from a targeted agent. Finally, it was unclear whether FGFR1 amplification or overexpression was a selective feature of either Lum-A or Lum-B tumors up until now. The characteristics usually associated with increased FGFR1 signaling (hormone resistance, increased metastatic potential, or increased cell replication [3, 17–21]) could lead to hypothesize that it might be an event mostly observed in Lum-B cases. Although the frequency of amplification was higher in Lum-B cases, we found in the H12O series that a significant percentage of Lum-A cases (6.6%). The findings were validated in the METABRIC Lum-A/B cohort, although the percentages of amplification were lower, in accordance with a generally better prognosis series (Table 1). In both cases, however, when the H12O and METABRIC series were split by the luminal subtypes, FGFR1 amplification or overexpression lost their statistical significance. We cannot think in a plausible biological reason that explains this phenomenon; rather, it is common to observe that when the sample sizes are reduced, it affects the statistical significance, particularly when it is not a highly penetrant factor. Similar results—albeit at a considerably higher frequency than for FGFR1 amplification—were found for FGFR1-mRNA overexpression (Fig. 1g). Unfortunately, since the samples of the METABRIC Lum-A/B are not currently accessible, RNAscope could not be validated in this dataset. The same phenomenon (loss of statistical power) was observed when the H12O was split by the luminal subtype to study the effects of RNAscope positivity in relapse-free survival. The definitive role of amplification and/or overexpression might require larger patient series. Similarly, the optimal cutoff point for defining FGFR amplification (Supplemental Figure 1) will be better addressed not only with larger patient series (since the number of patients with ratios of > 4 and > 6 is progressively smaller) but with the assessment of efficacy of FGFR1 inhibitors in ongoing trials stratified by FGFR1 copy number.

Our findings are potentially useful as well in order to define a potential therapeutic niche for FGFR1 inhibitors. Currently, most patients debut with metastatic disease after a number of years exposed to aromatase inhibitors. Our data show that cell lines chronically exposed to hormonal deprivation become resistant to this condition (Fig. 2b) and are cross-resistant to fulvestrant (Fig. 2c). During this process, MCF7 and T-47D upregulated FGFR1 at the transcriptomic and translational level (Fig. 2d). Both MCF7 and T-47D LTED variants with high FGFR1 were also resistant to the combination of hormones (DCC/fulvestrant) plus the CDK4/6 inhibitor palbociclib. The primarily amplified HCC1428 cell line was not fully resistant to DCC but it was resistant to fulvestrant and fulvestrant + palbociclib (Fig. 3a).

The fact that MCF7 and T-47D cell lines engineered to overexpress FGFR1 were as well resistant to fulvestrant and fulvestrant plus palbociclib, and parental MCF7/T-47D cells were not (Figs. 2c, f and 3a), suggest that FGFR1 overexpression or amplification is a condition that can be acquired during hormonal deprivation and leads to resistance to fulvestrant and fulvestrant plus palbociclib. The role of FGFR1 amplification (but not overexpression) in the acquisition of resistance to ribociclib plus hormonal treatment was already known [55]; however, it was unknown that such status could be achieved just after hormonal exposure. CDK4/6 inhibitors abrogate the entry in the S-phase by suppressing Rb phosphorylation; akin to Arteaga and Formisano [55], we found that the FGFR1-overexpressing variants did not suppress Rb phosphorylation and cell cycle completely (Fig. 4), unless rogaratinib was added to the drug combos. We found that the LTED-R variants also achieved maximum inhibition of Rb phosphorylation and cell cycle suppression in response to rogaratinib-containing combos (Fig. 4). Palbociclib plus fulvestrant was not sufficient to block Rb phosphorylation and abort the cell cycle, which had strong correlation with the colony assays (Fig. 3a and b) that showed that only the triplets can eradicate the growth of these models.

An additional point that deserves attention is that our data suggest that the percentage of patients with amplification and/or overexpression of FGFR1 in the metastatic setting might be quite high. Our study found that up to 24.5% of primary HR+ tumors display any of the two alterations. Since continuous hormonal deprivation seem to select for FGFR1 overexpression, it is quite likely that the percentage is higher in relapsed tumors after adjuvant aromatase inhibitors. This hypothesis requires a direct assessment in a collection of metastatic samples and constitutes a weakness of our study, since we did not check if the mentioned effect in vitro actually translates into a large percentage of patients switching from negative into positive in their metastatic lesions. Metastatic HR+ sample collections are usually characterized by several selection biases since usually clinicians sample metastatic lesions in case patients display atypical features. An unbiased sample cohort would be required to this end. Another weakness is that we did not find a convincing mechanism that explained why the lack of estrogens led to a stable overexpression of FGFR1. We did not find direct transcriptional repression of ER over the FGFR1 promoter (data not shown); however, the experiments shown in Supplemental Figure 2 suggest a simple sub-clonal selection of FGFR1-overexpressing variants. A definitive answer to this matter would require a single-cell RNAseq approach. Regardless, the ultimate mechanism is not relevant at this point since we just aim to justify a clinical trial with the triple combination in a specific population selected by the emerging phenotypic trait of hormonal deprivation: FGFR1 amplification or overexpression. We eagerly await the efficacy results of two trials combining erdafitinib, palbociclib, and fulvestrant (NCT03238196) and rogaratinib, palbociclib, and fulvestrant (NCT04483505) in advanced, hormonopositive, FGFR1-amplified breast cancer, which will also help to better understand the correlation between higher amplification rates of FGFR1 and rogaratinib/erdafitinib efficacy.

## Conclusions

Taken together, our data suggest that FGFR1 increase at the genomic or transcriptomic level drives the relapse of a significant proportion of hormone-positive patients. The fact that FGFR1 transcriptomic levels increase during hormonal deprivation and the increased risk of relapse observed in FGFR1-positive patients suggest that the proportion of FGFR1-positive patients (by FISH and/or RNAscope) in the metastatic patients could be considerably high. Since FGFR1 amplification and overexpression confer a similar risk, and their concordance is less than 70%, we believe that FGFR1 should be determined by combining both FISH and RNAscope in order to capture all potential candidates for FGFR1 inhibitors. Concerning the potential therapeutic niche for this drug class, given the widespread use of aromatase inhibitors during the adjuvant stage and in first-line metastatic setting, and considering the observed resistance and lack of complete cell cycle suppression in response to fulvestrant plus palbociclib, a triplet (fulvestrant plus palbociclib plus FGFR1 inhibition) could be studied in the second line setting. We have recently launched a clinical trial that will study the tolerability and preliminary efficacy of rogaratinib, fulvestrant, and palbociclib in FGFR1-positive (by RNAscope and/or FISH) HR+ breast cancer patients that have progressed to first line CDK4/6 inhibitor plus aromatase inhibitor in order to address the role of this driver of HR+ breast cancer aggressiveness.

## Supplementary Information

**Additional file 1.**

**Additional file 2.**

**Additional file 3.**

**Additional file 4.**

**Additional file 5.**

**Additional file 6.**

**Additional file 7.**

**Additional file 8.**

## Data Availability

The datasets used and/or analyzed during the current study are available from the corresponding author on reasonable request.

## Abbreviations

HRPBC: Hormone-positive breast cancer
FISH: Fluorescence in situ hybridization
LTED-R: Long-term estrogen deprivation
HR+: Hormone receptor-positive
Lum-A and Lum-B: Luminal A and luminal B
FGFR1: Fibroblast growth factor receptor 1
Rb: Retinoblastoma protein
HER2: Human epidermal growth factor 2
FBS: Fetal bovine serum
DCC: Dextran charcoal-stripped
