# Supplementary material for: FGFR1 amplification or overexpression and hormonal resistance in luminal breast cancer: rationale for a triple blockade of ER, CDK4/6, and FGFR1
Source: Breast Cancer Res. 2021 Feb 12;23:21. doi: 10.1186/s13058-021-01398-8 (PMC7881584; doi:10.1186/s13058-021-01398-8)
Supplement: Supplementary file 1 — Additional file 1. [file 13058_2021_1398_MOESM1_ESM.pdf]

Additional file 1: % Ki67 value, follow up time, relapse, luminal status, FGFR1:CEN8 FISH ratio, and RNAscope for each patient.

Patient	%Ki67	Follow up time (years)	Relapse (yes/no)	Luminal status	FGFR1:CEN8 ratio	RNAscope
27120174	48	3.96	0	B	1	0
27100230	8	5.49	0	A	1	1
27080199	5	4.99	0	A	N/A	N/A
27120231	10	5.06	0	A	1	1
27080250	16	4.96	0	B	1	1
27120232	5	4.98	0	A	1	3
27080256	15	5.01	0	B	N/A	N/A
27120224	10	4.84	0	A	N/A	N/A
27120295	55	4.58	0	B	1	N/A
27080311	20	4.63	0	B	1	1
27120279	15	1.59	1	B	1	1
27120227	5	4.52	0	A	1	1
27120228	20	4.48	0	B	1	1
27020375	5	4.32	0	A	1	1
27110011	30	4.29	0	B	1	0
27120233	17	4.39	0	B	1	N/A
27120234	20	4.53	0	B	1	N/A
27120235	30	5.04	0	B	1	2
27120236	5	4.38	0	A	N/A	N/A
27020497	18	4.16	0	B	1	N/A
27080459	4	4.08	0	A	1	N/A
27090440	18	4.08	0	B	1	N/A
27120239	1	3.53	0	A	1	N/A
27120240	15	4.01	0	B	1	0
27120241	5	3.83	0	A	N/A	1
27080514	3	3.91	0	A	N/A	2
27120242	7	4	0	A	1	2
27120287	3	3.58	0	A	1	N/A
27120294	45	3.66	0	B	1	N/A
27120288	15	3.75	0	B	1	N/A
27120243	10	4	0	A	1	1
27110129	20	4.17	0	B	1	2
27070004	10	5.87	0	A	1	1
27120244	10	1.15	0	A	1	0
27120245	10	6.36	0	A	1	1
27110060	20	6.17	0	B	1	3
27120246	1	6.15	0	A	1	1

27120247	15	3.97	0	B	6.25	4
27080060	50	6.18	0	B	1	1
27080087	20	6.12	0	B	5	3
27120248	10	5.9	0	A	1	N/A
27110058	20	5.96	0	B	1	2
27120249	12	5.54	0	A	N/A	N/A
27080113	4	3.6	1	A	1	0
27120250	10	4.8	0	A	1	1
27120251	25	3.49	0	B	1	1
27120285	60	0.35	1	B	N/A	N/A
27080246	55	1.03	1	B	1	N/A
27120226	10	2.42	1	A	1	1
27120278	15	1.58	1	B	1	N/A
27120290	45	2.99	1	B	1	N/A
27120304	33	1.17	1	B	N/A	3
27120283	31	5.76	1	B	1	N/A
27120297	25	5.76	1	B	1	3
27120229	5	4.79	0	A	1	1
27120280	5	1.08	0	A	1	0
27120238	10	2.08	0	A	N/A	N/A
7120872	8	10.98	0	A	1	1
7121157	10	8.72	0	A	1	2
7020664	9	10.62	0	A	1.16	2
7110215	13	13.68	0	A	1	2
7121124	2	11.63	0	A	1	0
7120878	6	10.62	0	A	1	0
7110320	1	10.31	0	A	N/A	N/A
7121119	4	11.8	0	A	1	0
7120621	6	10.52	0	A	1	1
7110337	7	11.29	0	A	1	N/A
7121146	5	9.6	0	A	1	N/A
7020949	5	9.91	0	A	N/A	2
7121149	12	9.23	0	A	1	0
7121118	8	11.84	0	A	N/A	N/A
7121127	10	10.89	0	A	N/A	N/A
7040660	3	8.26	0	A	1	1
7121153	2	8.63	0	A	1	0
7121134	3	9.95	0	A	N/A	N/A
7120877	9	10.54	0	A	1	1
7121141	10	10.01	0	A	1	N/A
7120889	4	5.18	0	A	1	1
7120879	6	11.19	0	A	1	N/A
7120888	2	5.51	0	A	1	N/A

7120861	9	14.19	0	A	1	N/A
7121116	3	12.34	0	A	1	1
7020303	5	10.18	0	A	1	1
7121117	10	9.28	0	A	1	1
7121111	10	12.2	0	A	N/A	3
7120898	5	8.15	0	A	1	N/A
7110264	7	11.88	0	A	N/A	N/A
7120871	4	10.93	0	A	1	0
7121123	9	11.84	0	A	1	1
7120859	7	12.94	0	A	N/A	0
7010856	3	11.37	0	A	N/A	0
7121133	12	12.8	0	A	1	N/A
7030768	12	9.2	0	A	1	1
7120882	9	10.6	0	A	1	1
7030230	2	9.79	0	A	1	4
7121110	10	12.33	0	A	1	N/A
7110345	12	12.04	0	A	1	0
7110360	5	11.56	0	A	1	1
7030174	13	9.66	0	A	1	2
7121135	13	10.86	0	A	N/A	N/A
7110284	3	12.52	0	A	1	N/A
7110376	9	10.24	0	A	1	0
7110285	9	12.23	0	A	1	N/A
7110286	11	12.47	0	A	1	N/A
7120862	0	12.25	0	A	1	2
7110220	7	11.1	0	A	1	N/A
7120863	7	11.13	0	A	N/A	N/A
7110343	5	7.29	0	A	1	0
7110206	7	10.54	0	A	1	N/A
7121140	1	10.06	0	A	1	0
7120883	8	10.52	0	A	N/A	N/A
7120876	2	12.88	0	A	1	2
7110265	13	12.55	0	A	N/A	N/A
7050752	10	4.69	0	A	N/A	0
7121128	7	8.34	0	A	1	1
7121120	3	11.96	0	A	1	1
7110193	3	8	0	A	1	1
7121136	9	10.18	0	A	1	N/A
7121139	11	10.58	1	A	6.25	N/A
7121132	6	10.74	0	A	1	1
7010122	10	1.25	0	A	1	1
7121129	4	11.1	0	A	1	0
7040643	9	4.08	1	A	1	2

7110282	4	1.65	1	A	1	1
7120881	4	4.02	1	A	N/A	2
7110271	6	4.06	1	A	1	N/A
7010678	6	1.73	1	A	N/A	1
7010097	6	6.58	1	A	N/A	0
7121126	5	7.23	1	A	1	0
7121145	6	9.28	0	A	1	N/A
7110396	9	7.61	1	A	1	N/A
7120885	20	1.61	1	B	5	4
7110189	13	3.02	1	A	1	N/A
7120855	11	2.03	1	A	N/A	4
7110188	13	7.18	0	A	1	N/A
7120858	10	7.44	1	A	1	N/A
7030672	10	8.51	0	A	1	2
7110205	12	5.81	1	A	4.5	N/A
7120870	5	11.26	1	A	N/A	N/A
7121144	13	5.95	1	A	1	N/A
7120850	8	4.78	1	A	1	0
7110202	5	9.82	1	A	N/A	0
7121125	30	11.43	0	B	N/A	3
7120474	32	9.82	0	B	2.2	1
7121159	20	8.38	0	B	2.66	3
7121161	34	8.21	0	B	1	1
7120864	15	2.4	0	B	1	0
7010908	25	11.33	0	B	1	1
7110362	17	11.26	0	B	1	1
7110414	35	10.38	0	B	1	3
7020516	30	10.88	0	B	2.5	4
7120895	70	3.86	0	B	1	1
7110393	65	11.45	0	B	1	2
7121109	25	12.15	0	B	1	2
7120893	17	3.67	0	B	1	N/A
7120894	10	6.34	0	A	N/A	N/A
7121155	20	8.87	0	B	1	0
7120887	16	5.36	0	B	1	1
7120890	32	4.96	0	B	1	N/A
7120886	20	5.32	0	B	1	N/A
7121138	60	10.27	0	B	1	2
7060855	20	3.87	0	B	1	1
7121158	20	8.36	0	B	N/A	N/A
7121156	13	8.53	0	A	1	N/A
7120896	20	1.56	0	B	1	0
7120892	28	4.66	0	B	1	1

7030724	20	9.34	0	B	1	N/A
7120860	13	12.13	0	A	1	2
7110240	30	11.1	0	B	1	N/A
7120868	27	3.6	0	B	1	1
7110346	15	12.78	0	B	1	1
7110325	15	10.23	0	B	1	0
7020975	17	10.35	0	B	1	1
7110268	15	9.18	0	B	2.5	4
7040576	17	5.67	0	B	N/A	N/A
7100551	16	12.86	0	B	1	0
7110322	90	12.98	0	B	1	0
7110374	17	10.4	0	B	N/A	2
7120884	22	10.06	0	B	1	N/A
7010873	35	11.28	0	B	1	1
7121112	20	10.13	1	B	N/A	N/A
7120875	30	13.64	0	B	1	0
7120880	15	10.43	0	B	N/A	2
7110389	17	10.55	0	B	4.5	4
7030426	15	11.14	0	B	1	0
7121142	20	10.14	0	B	1	2
7030710	14	9.36	0	B	5	4
7120873	17	11.09	0	B	1	1
7121113	20	4.08	0	B	1	0
7110324	13	10.21	0	B	1	0
7030406	14	6.99	0	B	N/A	N/A
7110385	27	8.16	0	B	1	N/A
7030375	35	9.68	0	B	3.87	0
7121121	19	0.21	1	B	1	1
7120891	17	6.34	0	B	N/A	2
7110294	20	8.13	1	B	1	0
7120869	16	10	1	B	1	1
7121143	15	6.01	1	B	5	N/A
7121122	14	8.67	1	A	7.5	4
7050260	28	5.31	1	B	5.175	4
7120897	15	2	1	B	N/A	0
7030457	50	1.55	1	B	3.75	N/A
7121130	40	1.55	1	B	5	N/A
7110379	23	7.35	0	B	1	0
7110382	20	8.05	1	B	N/A	0
7120865	20	3.32	1	B	5	4
7120857	20	1.61	1	B	1	2
7121114	15	2.87	1	B	1	0
7110272	22	1.64	1	B	N/A	3

7121137	30	4.03	1	B	1	1
7030483	30	4.99	1	B	N/A	3
7120866	20	1.47	1	B	1	N/A
7110256	41	0.98	1	B	N/A	3
7110287	20	4.52	1	B	1	N/A
7030423	20	2.55	1	B	N/A	N/A
7121154	30	0.12	1	B	N/A	N/A
7121152	20	4.61	1	B	1	N/A
7121160	60	4.64	1	B	1	2
7030492	40	0.13	1	B	N/A	0
7110209	6	9.56	1	A	2.66	0
7120849	20	3.39	1	B	1	1
7120867	26	2.25	1	B	1	1
7121150	24	3.45	1	B	N/A	0
7120856	18	7.36	1	B	3.33	3
7120854	17	10.15	0	B	1	2
7020932	15	0.67	1	B	1	3
7121148	40	6.4	1	B	1	1
7121131	17	8.35	1	B	1	1
7120373	33	3.3	1	B	1	0
7110245	20	4.76	1	B	1	1
7030419	10	11.35	0	A	2.2	4
7120874	27	4.25	1	B	1	N/A
7120853	42	1.76	1	B	1	0
7120851	18	2.82	1	B	1	N/A
7120852	48	1.88	1	B	5	4
7130051	8	6.71	1	A	N/A	1
7130050	10	13.75	1	A	N/A	N/A
7130049	3	15.22	1	A	4.5	N/A
7130048	13	11.62	1	A	N/A	N/A
7080691	30	3.58	1	B	1	0
7110323	31	10.89	1	B	N/A	N/A
7130042	30	1.49	1	B	N/A	0
7130052	24	8	1	B	1	2
7130054	45	7.88	1	B	1	3
7130044	1	6.23	1	A	1	0
7130053	8	8.04	1	A	N/A	1
7100171	30	2.17	1	B	1	2
7130043	20	0.33	1	B	N/A	N/A
7130045	25	4.12	1	B	N/A	N/A
7130046	53	5.23	1	B	1	0
7130047	7	5.41	1	A	1	N/A

N/A = not available due to sample quality issues
