# Supplementary material for: FGFR1 amplification or overexpression and hormonal resistance in luminal breast cancer: rationale for a triple blockade of ER, CDK4/6, and FGFR1
Source: Breast Cancer Res. 2021 Feb 12;23:21. doi: 10.1186/s13058-021-01398-8 (PMC7881584; doi:10.1186/s13058-021-01398-8)
Supplement: Supplementary file 2 — Additional file 2. [file 13058_2021_1398_MOESM2_ESM.pdf]

Additional file 2: Palbociclib and fulvestrant IC50 among the different cell lines.

Cell lines	IC_{50} Fulvestrant (nM)	IC_{50} Palbociclib (nM)
MCF7	0.20 ±0.02	51.9±0.010
MCF7-FGFR1	0.83 ±0.06	60.71±0.85
MCF7-LTED-R	1.03 ±0.07	80.91±6.58
T-47D	0.86 ±0.06	47.31±2.60
T-47D-FGFR1	2.24 ±0.08	66.37±8.19
T-47D-LTED-R	1.49 ±0.02	68.84±3.88
HCC1428	13.95 ±0.69	*
HCC1428-LTED-R	18.75 ±1.19	*

*Not reached at 500nM

IC_{50} Values are mean±SEM of three independent experiments (n=3).
