# Supplementary material for: FGFR1 amplification or overexpression and hormonal resistance in luminal breast cancer: rationale for a triple blockade of ER, CDK4/6, and FGFR1
Source: Breast Cancer Res. 2021 Feb 12;23:21. doi: 10.1186/s13058-021-01398-8 (PMC7881584; doi:10.1186/s13058-021-01398-8)
Supplement: Supplementary file 3 — Additional file 3. [file 13058_2021_1398_MOESM3_ESM.pdf]

**A**

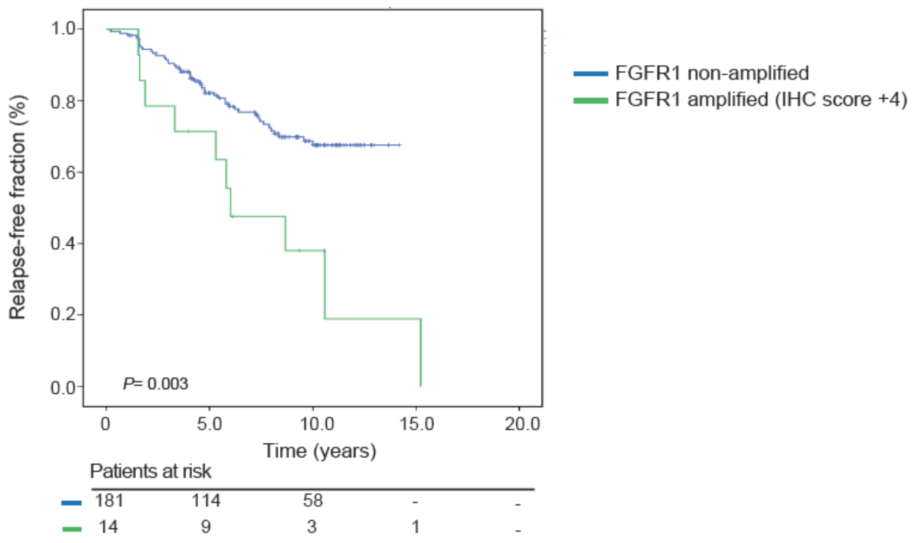


**B**

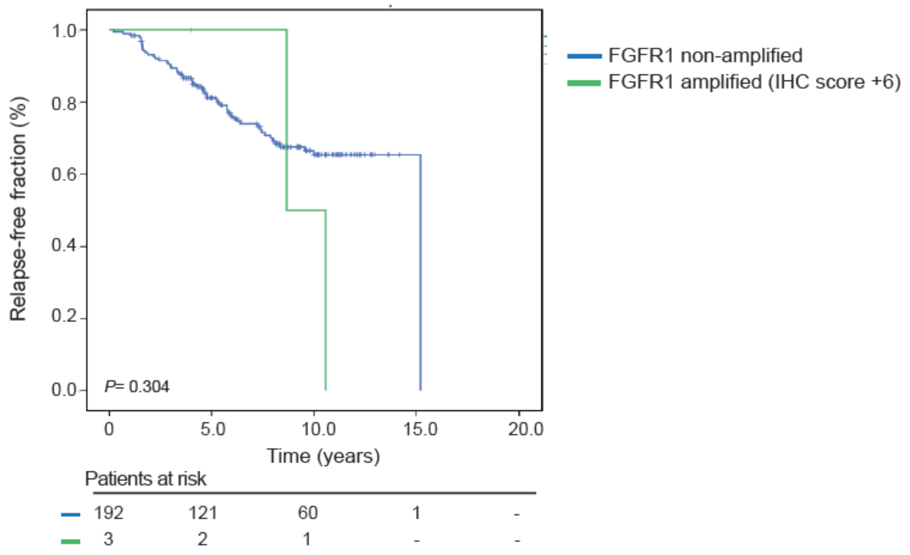


**Supplementary Fig. 1: Analysis applying different FGFR1/centromere ratios for amplification.**

**A**. Ratio >4: non-amplified cases: median relapse-free survival not reached (95% CI: not reached – not reached); average relapse-free survival 11.2 years (95% CI: 10.5 – 11.9); amplified cases: median relapse-free survival 6.0 years (95% CI: 1.57 – 10.44); *P* = 0.003. Hazard ratio for relapse: 2.8 (95% CI: 1.38 – 5.74). **B**. Ratio > 6: non-amplified cases: median relapse-free survival 15.2 years (95% CI: 11.2 – 17.4); amplified cases: median relapse-free survival: 8.7 (95% CI: 2.42 – 12.1); *P* = 0.30. Hazard ratio for relapse: 2.0 (95% CI: 0.50 – 8.47).
