# Supplementary material for: FGFR1 amplification or overexpression and hormonal resistance in luminal breast cancer: rationale for a triple blockade of ER, CDK4/6, and FGFR1
Source: Breast Cancer Res. 2021 Feb 12;23:21. doi: 10.1186/s13058-021-01398-8 (PMC7881584; doi:10.1186/s13058-021-01398-8)
Supplement: Supplementary file 4 — Additional file 4. [file 13058_2021_1398_MOESM4_ESM.pdf]

Supplemental Figure 2

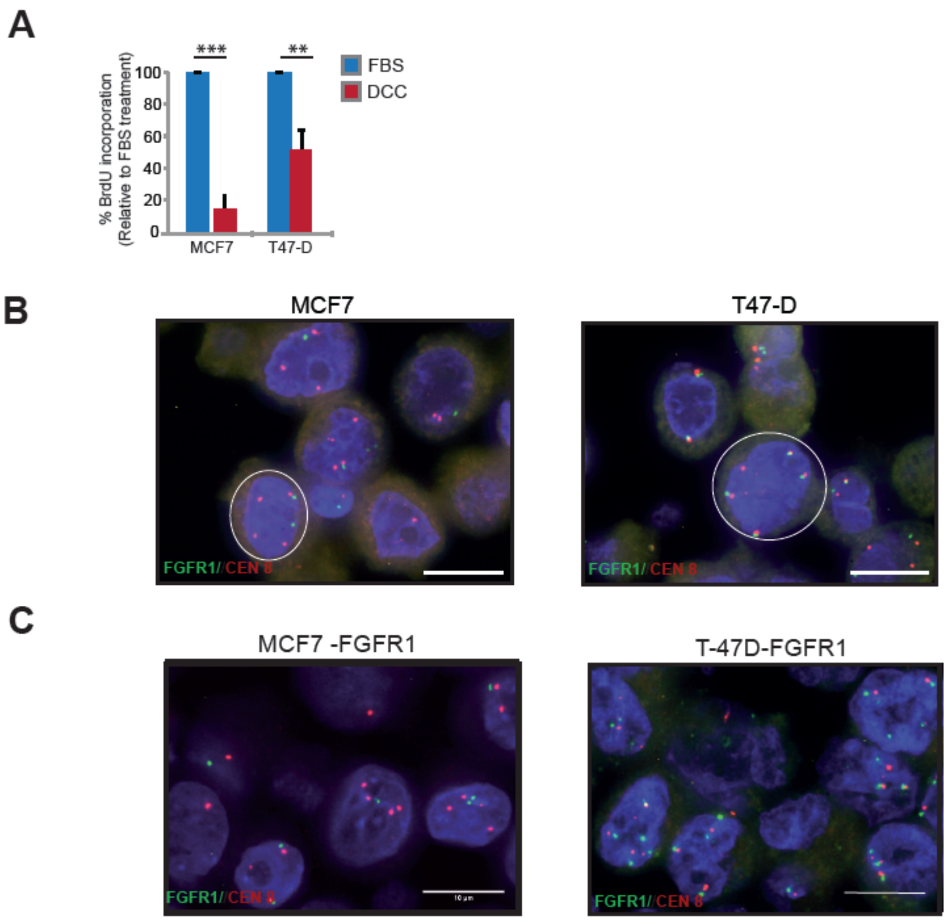

Supplemental Figure 2: Limited replication during the acquisition of the LTED-R phenotype. (A) MCF7 and T47-D were cultured for 48 hours deprived from estrogens in DCC media prior to measure the changes in BrdU incorporation, and compared to the same cells growing in full media. It can be appreciated how MCF7 almost suppressed replication, whereas T47-D cells maintained approximately 50% of their replication rate. **(B)** FGFR1 FISH in regular MCF7 and T47-D prior to undergo the 2-year selection rounds of LTED-R variants. Isolated polysomic cells can be recognized (i.e., circled cells harbor 4-5 copies of each FGFR1 and chromosome 8 centromere – despite the increased number of copies, these cells are not amplified because the gene:centromere ratio is 1:1). Such polysomic cells are surrounded by cells with regular copy numbers. When LTED-R variants are analyzed, the number of polysomic cells is larger and the transcriptional and protein levels of FGFR1 levels are globally higher, suggesting that such cells are expanded during the selection process. **(C)** FGFR1 FISH in the engineered FGFR1-overexpressing variants (T-47D-FGFR1 and MCF7-FGFR1).
