# Supplementary material for: FGFR1 amplification or overexpression and hormonal resistance in luminal breast cancer: rationale for a triple blockade of ER, CDK4/6, and FGFR1
Source: Breast Cancer Res. 2021 Feb 12;23:21. doi: 10.1186/s13058-021-01398-8 (PMC7881584; doi:10.1186/s13058-021-01398-8)
Supplement: Supplementary file 5 — Additional file 5. [file 13058_2021_1398_MOESM5_ESM.pdf]

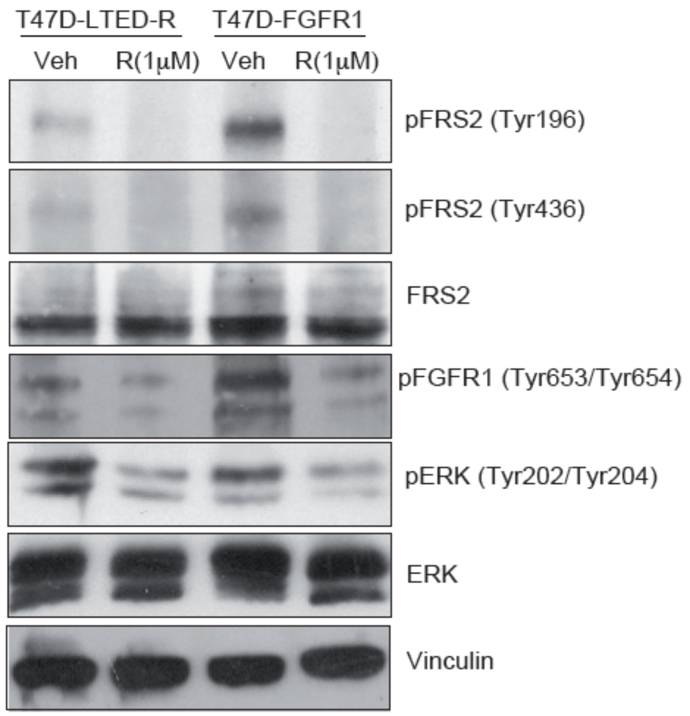

Supplementary Fig. 3: Pharmacodynamic effect of rogaratinib in T-47D variants. T-47D-LTED-R and FGFR1-overexpressing variants were exposed to pre-incubation with vehicle (Veh) or rogaratinib (R) for 6 hours followed by stimulation with bFGF for 10 minutes (20 ng/ml). It can be appreciated how rogaratinib suppresses phosphorylation of ERK, FGFR1 and FRS2 proteins.
