# Supplementary material for: FGFR1 amplification or overexpression and hormonal resistance in luminal breast cancer: rationale for a triple blockade of ER, CDK4/6, and FGFR1
Source: Breast Cancer Res. 2021 Feb 12;23:21. doi: 10.1186/s13058-021-01398-8 (PMC7881584; doi:10.1186/s13058-021-01398-8)
Supplement: Supplementary file 6 — Additional file 6. [file 13058_2021_1398_MOESM6_ESM.pdf]

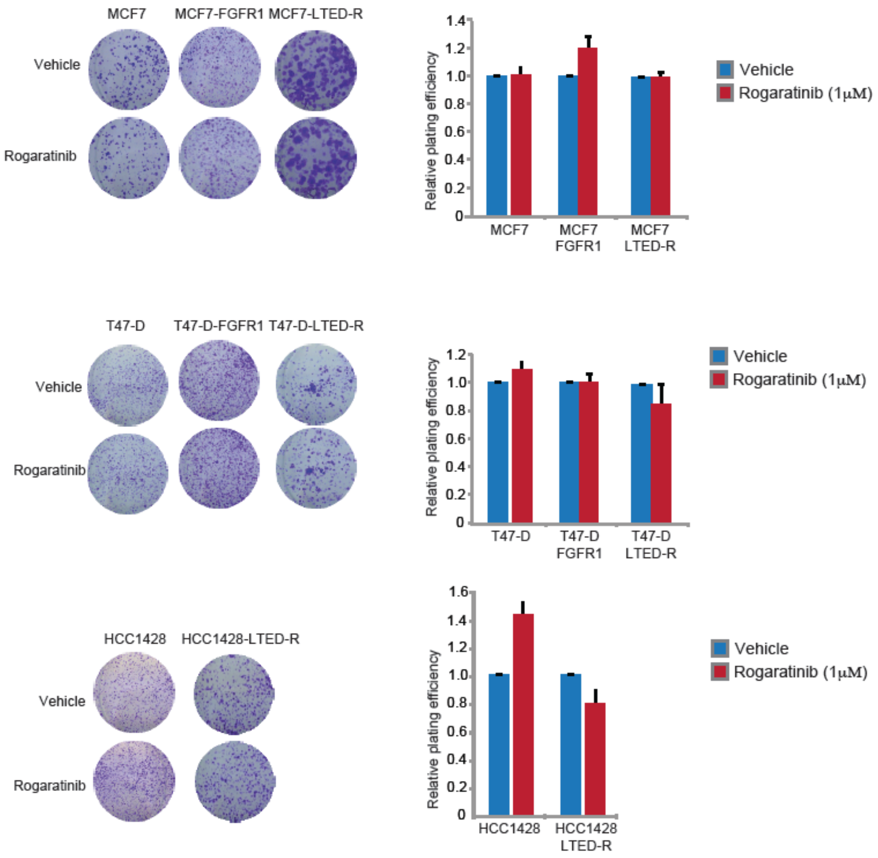

Supplementary Fig. 4: Lack of efficacy of rogaratinib monotherapy. MCF7, T47-D, HCC1428 and their LTED-R variants were exposed for >1 week to rogaratinib (1μM). FGFR1-overexpressing MCF7 and T-47D variants were tested as well. The relative plating efficiency was not affected by rogaratinib monotherapy regardless of the FGFR1 status.
