# Supplementary material for: FGFR1 amplification or overexpression and hormonal resistance in luminal breast cancer: rationale for a triple blockade of ER, CDK4/6, and FGFR1
Source: Breast Cancer Res. 2021 Feb 12;23:21. doi: 10.1186/s13058-021-01398-8 (PMC7881584; doi:10.1186/s13058-021-01398-8)
Supplement: Supplementary file 7 — Additional file 7. [file 13058_2021_1398_MOESM7_ESM.pdf]

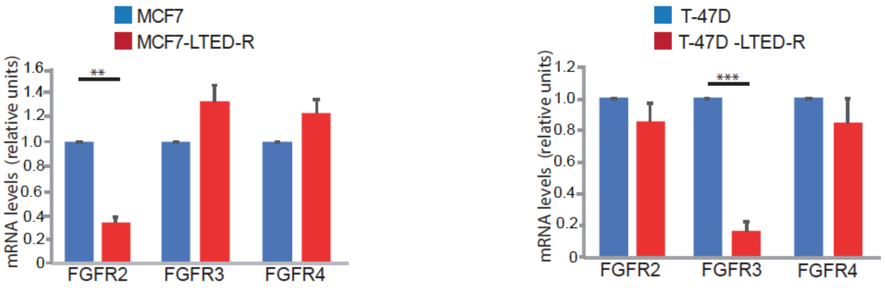

Supplementary Fig. 5: Expression levels of FGFR2, 3 and 4 mRNA among parental and LTED variants. As opposed to FGFR1 mRNA, which seems to be consistently increased in hormonal-resistant models, the other isoforms do not show a congruent pattern in such models, what precludes at this moment any causal interpretation of their role in the hormone-resistant phenotype.
