# Supplementary material for: FGFR1 amplification or overexpression and hormonal resistance in luminal breast cancer: rationale for a triple blockade of ER, CDK4/6, and FGFR1
Source: Breast Cancer Res. 2021 Feb 12;23:21. doi: 10.1186/s13058-021-01398-8 (PMC7881584; doi:10.1186/s13058-021-01398-8)
Supplement: Supplementary file 8 — Additional file 8. [file 13058_2021_1398_MOESM8_ESM.pdf]

Supplemental Figure 6

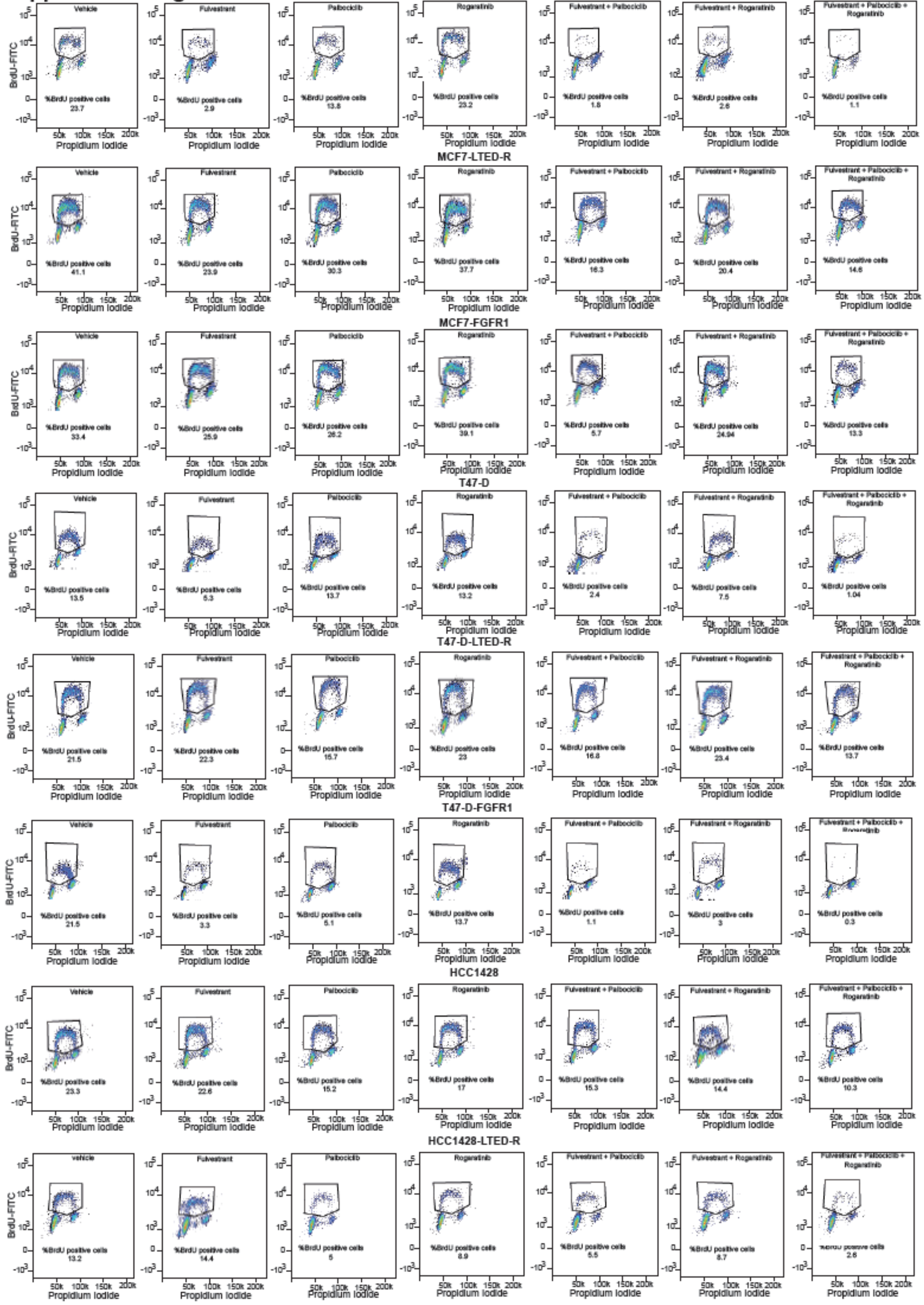

Supplementary Fig. 6: Effects of rogaratinib-containing combinations in the cell cycle of LTED-R FGFR1-overexpressing or amplified cell lines. Cell cycle charts of the different *in vitro* models in presence of vehicle or drug (monotherapy or combinations) for 48h. 1 µM rogaratinib, 0.5 nM fulvestrant (MCF7 cell lines) or 1.5 nM (T47-D and HCC1428 cell lines) and 50 nM (MCF7) or 100 nM (T47-D and HCC1428) palbociclib. Gated areas: percentage of BrdU-positive cells.
